# EHMTI-0360. Chronic sympathetic activation in migraine headache: unique to migraine or common to sympathetic nervous system disorders?

**DOI:** 10.1186/1129-2377-15-S1-J12

**Published:** 2014-09-18

**Authors:** MR Thomas

**Affiliations:** 1 Clinical Psychology, Private Practice, Whitby, Canada

## 

The mechanisms of autonomic nervous system dysfunction in migraine are not well understood. It has been proposed that chronic/excessive SNS activation contributes to migraine episodes by rapidly depleting norepinephrine stores while increasing the release of dopamine, adenosine triphosphate, adenosine and prostaglandins. Research showing significantly colder hands in female migraineurs than healthy controls between headaches suggests evidence of chronic/excessive SNS activation in female migraineurs.

A recent audit of standardized clinical assessment data collected over several years during a 26 minute psychophysiological mental stress assessment revealed interesting results which shed light on this dilemma. Data included hand skin temperature (HST), frontal sEMG, HR, SCR, respiration rate and the Anxiety Sensitivity Index. Ten female migraineurs (MH), 10 females with muscle contraction headache (MCH) and 10 females with panic disorder (PD) were compared.

MCH controlled for the effects of having a distressing non-migraine/non SNS headache disorder. PD controlled for the effects of having a distressing non-headache, SNS disorder.

There were no significant between-group differences for age and most test scores.

A repeated measures ANOVA showed a significant between-groups difference for HST. The MCH group was in the normal range while the MH and PD groups were well below normal and significantly colder than the MCH group.

The MH group results suggest chronic SNS arousal. However the PD group's similar result and the MCH normal result suggests that chronic SNS arousal is a characteristic of SNS disorders and is not unique to migraine. Alone, it appears insufficient to explain migraine episodes. Additional factors are proposed.

No conflict of interest.

